# Single-Cell RNA Sequencing of Hematopoietic Stem and Progenitor Cells Treated with Gemcitabine and Carboplatin

**DOI:** 10.3390/genes11050549

**Published:** 2020-05-14

**Authors:** Niclas Björn, Ingrid Jakobsen, Kourosh Lotfi, Henrik Gréen

**Affiliations:** 1 Clinical Pharmacology, Division of Drug Research, Department of Biomedical and Clinical Sciences, Linköping University, 581 85 Linköping, Sweden; niclas.bjorn@liu.se (N.B.); ingrid.jakobsen@regionorebrolan.se (I.J.); kourosh.lotfi@liu.se (K.L.); 2 Department of Laboratory Medicine, Örebro University Hospital, 701 85 Örebro, Sweden; 3 Department of Hematology, Linköping University Hospital, 581 85 Linköping, Sweden; 4 Department of Forensic Genetics and Forensic Toxicology, National Board of Forensic Medicine, 587 58 Linköping, Sweden

**Keywords:** hematopoietic stem and progenitor cells, single-cell RNA sequencing, gemcitabine, carboplatin, myelosuppression, toxicity, adverse drug reactions

## Abstract

Treatments that include gemcitabine and carboplatin induce dose-limiting myelosuppression. The understanding of how human bone marrow is affected on a transcriptional level leading to the development of myelosuppression is required for the implementation of personalized treatments in the future. In this study, we treated human hematopoietic stem and progenitor cells (HSPCs) harvested from a patient with chronic myelogenous leukemia (CML) with gemcitabine/carboplatin. Thereafter, scRNA-seq was performed to distinguish transcriptional effects induced by gemcitabine/carboplatin. Gene expression was calculated and evaluated among cells within and between samples compared to untreated cells. Cell cycle analysis showed that the treatments effectively decrease cell proliferation, indicated by the proportion of cells in the G2M-phase dropping from 35% in untreated cells to 14.3% in treated cells. Clustering and t-SNE showed that cells within samples and between treated and untreated samples were affected differently. Enrichment analysis of differentially expressed genes showed that the treatments influence KEGG pathways and Gene Ontologies related to myeloid cell proliferation/differentiation, immune response, cancer, and the cell cycle. The present study shows the feasibility of using scRNA-seq and chemotherapy-treated HSPCs to find genes, pathways, and biological processes affected among and between treated and untreated cells. This indicates the possible gains of using single-cell toxicity studies for personalized medicine.

## 1. Introduction

The two chemotherapeutic drugs gemcitabine and carboplatin are used in the treatment of many cancer types. Treatments that include these two drugs are harsh, and are often associated with adverse drug reactions (ADRs) [1,2,3]. The dose-limiting ADRs are mainly the hematological toxicities neutropenia, leukopenia, and thrombocytopenia. Even if doses are adjusted before administration, there is considerable variability in hematological ADRs among patients; some show no signs of toxicity, while others experience life-threatening levels of hematological toxicity [4,5,6,7]. Understanding the development of these hematological toxicities is important and necessary for the implementation of more personalized treatment approaches in which doses are adjusted according to the patient’s risk of toxicity before treatment.

Classical chemotherapy drugs have nonspecific mechanisms of action and generally attack rapidly dividing cells, which is an underlying component of the associated toxicities induced by the drugs. Human bone marrow with its rapid development of blood cells consequently comes under severe attack, which leads to a reduced amount of mature blood cells. There are studies that explore hematopoietic stem and progenitor cells (HSPCs) and/or bone-marrow-derived cells using bulk transcriptome analysis [8,9,10]. The recent developments of single-cell analyses, including single-cell RNA sequencing (scRNA-seq) reviewed in [11], have shown that there is more information to obtain from cells than the average expression seen in bulk samples [12,13,14,15,16,17]. However, how the HSPCs of human bone marrow are affected on a transcriptional level by gemcitabine and carboplatin and which biological processes and pathways are of importance is largely unknown and unstudied.

In this study, we had the rare opportunity to analyze HSPCs selected based on CD34^+^ in a clinical stem cell harvest. The HSPCs were then exposed to both gemcitabine and carboplatin in vitro before scRNA-seq analysis. This is not only one of the few analyses using this cell type, but, to our knowledge, also the first study that evaluates the feasibility of using the scRNA-seq of HSPCs to determine effects induced by chemotherapeutic treatments.

## 2. Materials and Methods

### 2.1. Patient Sample

The use of human HSPCs in this study was approved by the regional ethics committee in Linköping, Sweden (DNR 2017/384-31), and the patient gave written informed consent, as per the Declaration of Helsinki, before inclusion. The patient had previously gone through a stem cell harvest for a possible autologous stem cell transplantation, which was part of the standard treatment protocols for chronic myelogenous leukemia (CML) at the time. However, these HSPCs were never used in the patient’s treatment of CML, because of the success of the updated treatment guidelines including the use of tyrosine kinase inhibitors. The harvest was performed with the CliniMACS^®^ CD34 Reagent System (Miltenyi Biotec, Bergisch Gladbach, Germany) at Linköping University Hospital (Linköping, Sweden), and the HSPCs were subsequently cryopreserved.

### 2.2. Cell Culture and Treatments

The cryopreserved HSPCs were thawed in a 37 °C water bath and washed with pure RPMI 1640 (Gibco, Life Technologies, Paisley, UK). After this, the cells were cultured at high density (1 million cells/mL) to acclimatize after thawing in tissue-culture-treated (TC-treated) T-25 flasks using StemMACS HSC Expansion Media XF, human (Miltenyi Biotec) supplemented with StemMACS HSC Expansion Cocktail, human (Miltenyi Biotec), and kept at 37 °C in a humidified atmosphere containing 5% CO_2_ for 48 h.

Gemcitabine (Toronto Research Chemicals, Toronto, Canada), carboplatin (Toronto Research Chemicals), or no drug (as a control) were used for the MTT assay (Molecular Probes, Life Technologies, Paisley, UK) to derive the appropriate drug concentrations for the scRNA-seq experiments. The MTT was mainly carried out as previously described [18], however with some changes. Briefly, 100 µL of culture media with 3 × 10^5^ cells/mL was added to a TC-treated 96-well-plate were nine different concentrations of gemcitabine and carboplatin in triplicates had been respectively added as 5 µL and 10 µL dilutions in sterile-filtered Milli-Q^®^ to yield the final concentrations listed in Table 1. Triplicate wells where cell suspension was added to the respective volumes of sterile-filtered Milli-Q^®^ without any drugs were used as controls. Triplicates of media without any cells were added to the respective volumes of sterile-filtered Milli-Q^®^ without any drugs were used as blanks. Following 24 h of incubation 10 µL of MTT (5 mg/mL) was added to each well after which the plates were incubated for another 4 h. Then the formazan salt crystals were dissolved with the addition of 100 µL solution of 10% SDS with 0.01 M HCL to each well and incubated overnight. Lastly, the VersaMax ELISA Microplate Reader (Molecular Devices LLC, San Jose, CA, USA) was used for measuring the absorbance at 580 nm, the blank was subtracted from the other measurements. The absorbance was then normalized to the plate controls representing 100% viability. From this the half-maximal inhibitory concentration (IC_50_)-values with 95% confidence intervals (CI) were calculated using three parameters non-linear curve fits in GraphPad Prism version 8.3.0 for Windows (GraphPad Software, La Jolla, CA, USA).

For the actual drug treatments and scRNA-seq experiment, the HSPCs were thawed and acclimatized in media for 48 h, as above. Then dead cells were removed using the Dead Cell Removal Kit (Miltenyi Biotec) following the manufacturer’s instructions. After this, the HSPCs were seeded at a concentration of 5 × 10^5^ cells/mL in TC-treated 6 well-plates using a volume of 2 mL/well. The cells were subsequently treated for 24 h under four different conditions: Carboplatin High—treatment with 150 µg/mL; Carboplatin Low—treatment with 18.75 µg/mL; Gemcitabine—treatment with 25 ng/mL; and Control—100 µL sterile-filtered Milli-Q^®^ (since the drugs were diluted in sterile-filtered Milli-Q^®^ and added as 100 µL). These final treatment conditions were selected based on the results from the MTT assay (see the results Section 3.1—Patient Characteristics and the MTT Assay).

### 2.3. Single-Cell RNA Sequencing

After the 24 h treatments, the samples were concentrated to roughly 2500 cells/µL in sterile-filtered PBS supplemented with 0.1% BSA (Sigma-Aldrich, St. Louis, MS, USA) and vigorously pipetted to ensure a single-cell suspension (visually confirmed). From the four samples, we extracted single cells and prepared sequencing libraries using the ddSEQ™ Single-Cell Isolator (Bio-Rad, Hercules, CA, USA) together with the SureCell™ Whole Transcriptome Analysis 3′ Library Prep Kit (Illumina, San Diego, CA, USA) following the manufacturers’ protocols. The sequencing-ready libraries were then sequenced on the NextSeq 500 System (Illumina) using the NextSeq 500/550 High Output Kit v2.5 150 Cycles (Illumina) according to the manufacturer’s instructions.

### 2.4. Alignment and Gene Expression

FASTQ files with the raw sequencing data were downloaded from Illumina’s BaseSpace Sequence Hub. Then the ddSeeker [19] and Drop-seq [20] protocols for processing scRNA-seq data were followed. Briefly, ddSeeker version 0.9.0 was used to combine the R1 and R2 raw sequencing read files into unmapped binary alignment map (uBAM) files tagged with cell barcodes and unique molecular identifiers (UMI). The data was sequenced in four lanes, and uBAMs from separate lanes for the same sample were merged with cat in SAMtools version 1.9. The uBAM files were subsequently queryname sorted with SortSam in Picard Tools version 2.20.3 before reads with barcode sequencing errors were filtered out using FilterBAM in Drop-seq version 2.3.0. The uBAM files were then converted back to FASTQ files with SamToFastq in Picard Tools before they were aligned to the human reference genome GRCh38.77 using STAR version 2.7.1a [21]. The alignment BAM files were sorted with SortSam in Picard Tools and then merged with the cell and UMI-tagged uBAMs using MergeBamAlignment in Picard Tools. The alignments were then tagged with genes using TagReadWithGeneFunction in Drop-seq. BamTagHistogram in Drop-seq was then used to extract the number of reads per cell barcode and from this, the number of cells to extract from each of the four samples could be determined. The gene expression for these cells was lastly determined with DigitalExpression in Drop-seq.

### 2.5. Data Analyses with the Seurat R Package

R version 3.6.1 [22] was used along with the R toolkit Seurat version 3.0.2 [23,24] for single-cell genomics to further analyze the gene expression of the four samples.

#### 2.5.1. Filtering, Normalization, Highly Variable Genes, and Cell Cycles

Only the expression of genes with reads in at least 3 cells were kept for each sample and used as input for Seurat. Then, based on the Seurat guidelines, the overall filtering criteria to keep high-quality cells were determined to percent mitochondrial reads/cell < 10%, reads/cell < 13,500, and 200 < genes/cell < 4750. The gene expression of high-quality cells was thereafter normalized according to Equation 1, as implemented in the default Seurat function NormalizeData. From the normalized data, the 2000 most variable gene features for each sample were determined with the function FindVariableFeatures in Seurat. Focusing on the highly variable gene features in downstream single-cell analyses helps to highlight biological signals [23,24,25]. To determine in which phase of the cell cycle all high-quality cells were, we used the Seurat function CellCycleScoring with the default settings which utilizes the cell cycle markers suggested by Kowalczyk et al. [26] and Tirosh et al. [27], 43 and 54 genes for S and G2M phase, respectively (all of which are listed in Table S5 of Tirosh et al. [27]). The CellCycleScoring function basically assigns a score for each cell based on the expression of these genes and in principal the S and G2M genes should have anticorrelated expression and cells without expression are deemed to be in G1.

(1) $$\mathrm{Normalized}\;\mathrm{expression}=\ln\left(1+\frac{\mathrm{gene}\;\mathrm{expression}\times 10^{4}}{\mathrm{total}\;\mathrm{cell}\;\mathrm{expression}}\right),$$

#### 2.5.2. Dimensionality Reduction, Clustering, and Differentially Expressed Genes

The dimensional reduction technique, principal component analysis (PCA), was performed on linearly transformed (scaled) data for the 2000 most variable genes using RunPCA in Seurat. 

Thereafter, clustering was done using the 20 first principal components (PCs) and the Seurat functions FindNeighbors and FindClusters. To visualize the clustering, we implemented the nonlinear dimensionality reduction techniques t-distributed stochastic neighbor embedding (t-SNE) [28] and uniform manifold approximation and projection (UMAP) [29] in the Seurat functions RunTSNE and RunUMAP.

Differential gene expression between cells within clusters and between clusters was determined using the function FindMarkers, in Seurat with the criteria that genes had to be present in at least 30% of the cells.

We also combined the treated cells and control cells in integrated analyses which promotes comparative analyses of scRNA-seq data from different samples. This enables us to compare, identify, and visualize both similarities and uniqueness in the HSPCs response to the chemotherapy exposure when compared to their untreated control. The Seurat functions FindIntegrationAnchors and IntegrateData were used for integrating and combining the treated and control datasets before scaling, PCA, clustering, and differential expression analyses were performed as explained above.

### 2.6. Gene Set Enrichment Analyses

The R package clusterProfiler version 3.12.0 [30] was used for gene set enrichment analyses of KEGG pathways and Gene Ontologies (GOs).

### 2.7. Data Availability

The sequencing data that support the findings in the study have been deposited at the European Genome-phenome Archive (EGA), which is hosted by the EBI and the CRG, under accession number EGAS00001004381.

## 3. Results

### 3.1. Patient Characteristics and the MTT Assay

**Figure 1:**
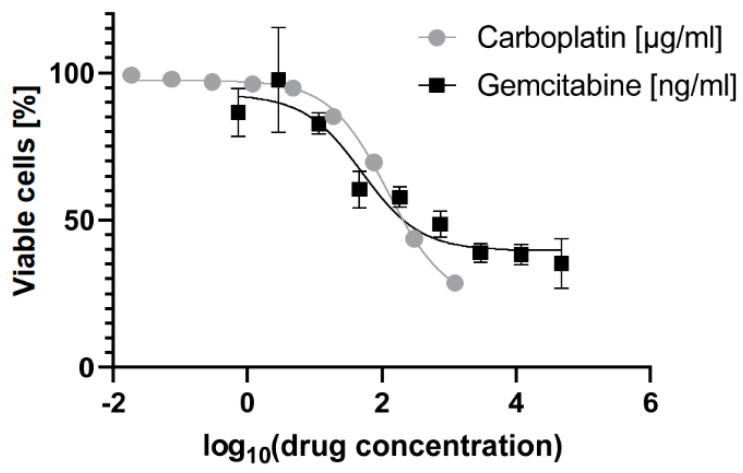
The dose-response curves determined using the MTT assay of the hematopoietic stem and progenitor cells (HSPCs) treated for 24 h with gemcitabine in log_10_(ng/mL) and carboplatin in log_10_(µg/mL). The error bars denote the standard deviation.

The HSPCs used in this study were donated by a female CML patient above 50 years of age with a high-risk (≥1481) Hasford score [31] at the time of harvest. From the initial MTT assay of the HSPCs, visualized in Figure 1, the IC_50_-values were determined: for carboplatin, 123.4 µg/mL (95% CI = 109.8–138.7 µg/mL); and for gemcitabine, 51.1 ng/mL (95% CI = 21.8–138.9 ng/mL). The final treatment conditions were chosen from these results: Carboplatin High 150 µg/ml, just above the range of IC_50_; for Carboplatin Low 18.75 µg/mL, a mild treatment; and for Gemcitabine 25 ng/mL, in the lower range of the IC_50_.

### 3.2. scRNA-seq Alignment and Gene Expression

The sequencing outputted a total of 120 million reads of which 74% mapped uniquely to the reference genome. Initial filtering of cells was done based on the data from BamTagHistogram in Drop-seq, visualized in Figure S1. This yielded a total of 1475 cells for which gene expression was determined, see Table S1.

### 3.3. High-Quality Cells, Most Variable Genes, and Cell Cycle Analysis

**Figure 2:**
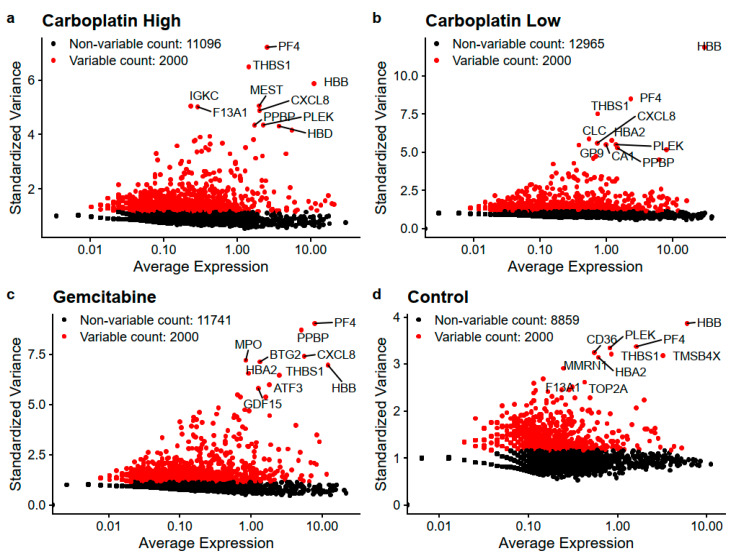
Depicts the most variable genes for (**a**) Carboplatin High, (**b**) Carboplatin Low, (**c**) Gemcitabine, and (**d**) Control. The ten genes with the highest standardized variance are written out for each sample.

**Figure 3:**
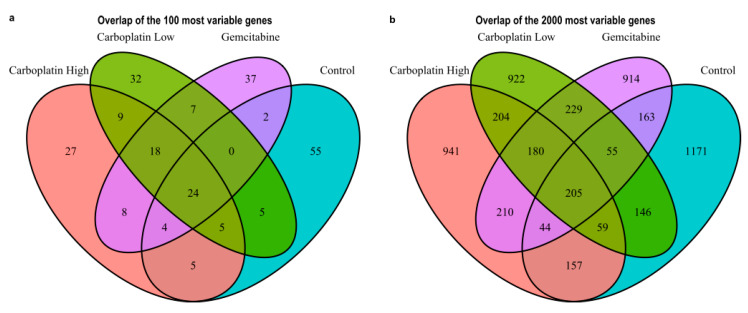
Venn diagrams showing the overlap between the treatment conditions Carboplatin High, Carboplatin Low, Gemcitabine, and Control. (**a**) The top 100 and (**b**) the top 2000 most variable genes.

The overall filtering of cells to keep high-quality cells is visualized in Figure S2 and in the end, this yielded a total of 1172 high-quality cells with reads mapping to 15,832 unique genes. Table 2 lists the number of cells, genes/cells, and the number of cells with reads detected for the genes CD34, ABL1, and BCR to get an understanding of the expression of HSPC and CML markers in high-quality cells. From these cells, the most variable genes were determined for Carboplatin High, Carboplatin Low, Gemcitabine, and Control cells, as visualized in Figure 2. The top 25 most variable genes for each sample are listed in Table 3. The overlaps of variable genes are visualized using Venn diagrams of the top 100 and 2000 most variable genes for each sample in Figure 3a,b, respectively. In total the top 100 and 2000 genes for each sample represent 238 and 5600 unique genes, respectively. Further, the Venn diagrams make it clear that of the top 100 genes, 27%–55% are within just one of the samples and 24% shared by all, whereas for the top 2000 about half are unique to each sample and the other half are shared among at least two samples and 10% are shared by all. This tells us that a lot of the variability in gene expression changes with the treatments which indicate that different effects are induced by the treatments, but we also see that a lot of the gene expression is shared which is also expected as it all stems from the same patient samples HSPCs.

**Figure 4:**
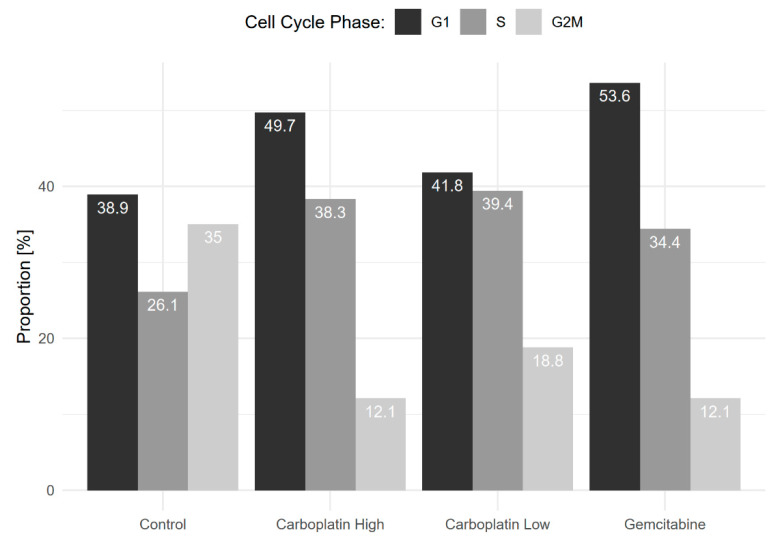
Shows the proportion of cells in G1 (Gap 1), S (Synthesis), and G2M (Gap 2 and Mitosis) phases of the cell cycle for each sample. A clear decrease in the proportion of cells in G2M for the chemotherapy-treated samples compared to the control was seen.

Cell cycle phase analysis was subsequently carried out using computational inference, see Figure 4. The figure shows a clear decrease in the proportion of cells in the G2M phase for the chemotherapy-treated samples, on average, 14.3%, compared to 35% for the control cells. This sanity-check shows that the chemotherapeutic treatments lead to decreased proliferation, as they should. 

### 3.4. Separate Analysis of Samples

**Figure 5:**
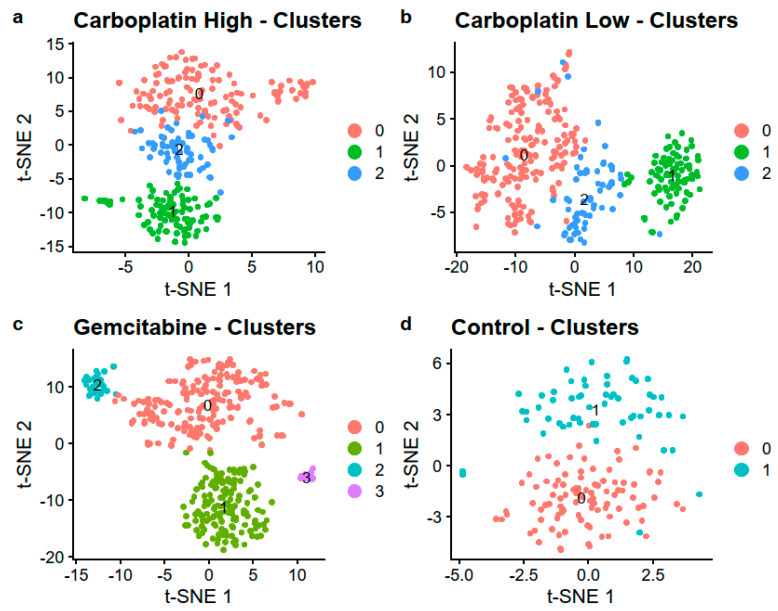
Visualization using t-SNE colored by the identified clusters in the four samples (**a**) Carboplatin High, (**b**) Carboplatin Low, (**c**) Gemcitabine, and (**d**) Control.

No clusters formed on the first two PCs of the four samples, see Figure S3. By utilizing the first 20 PCs, we were able to identify 3, 3, 4, and 2 clusters for Carboplatin High, Carboplatin Low, Gemcitabine, and Control, respectively, as visualized with t-SNE in Figure 5 and UMAP in Figure S4. The number of cells in each cluster is listed in Table 4. Differential gene expression that compared the different clusters with their respective cluster 0 showed, on average, 630 differentially expressed genes after adjusting for multiple testing using the Benjamini Hochberg method, all listed in Table S2. 

The KEGG and GO enrichment analysis of the differentially expressed genes shows that the genes that are differentially expressed between clusters are related to specific pathways and ontologies. Meaning that the clusters within the samples have differences in which pathways and GOs that are active and regulated because of effects induced by the treatments. This showcases that a single-cell approach on treated and control HSPCs can be used to find cellular changes and responses affected by the treatments. All KEGG pathways and GOs with adjusted (Benjamini Hochberg) *p*-value < 0.05 are listed in Table S3. The difference between cluster 1 and 0 in the control sample seems to be attributable to differences in mRNA, endoplasmic reticulum, and translation with significant GOs, including “protein localization to endoplasmic reticulum”, “translational initiation”, “mRNA catabolic process”, “protein targeting to ER”, “translation”, and “RNA catabolic process”.

### 3.5. Integrated Analysis of Treated and Control Samples

We then wanted to see if we could distinguish differences between treated and control cells. For this purpose, we integrated and merged the cells into Carboplatin High vs. Control, using the cells from Carboplatin High and Control; Carboplatin Low vs. Control, using the cells from Carboplatin Low and Control; and Gemcitabine vs. Control, using the cells from Gemcitabine and Control.

**Figure 6:**
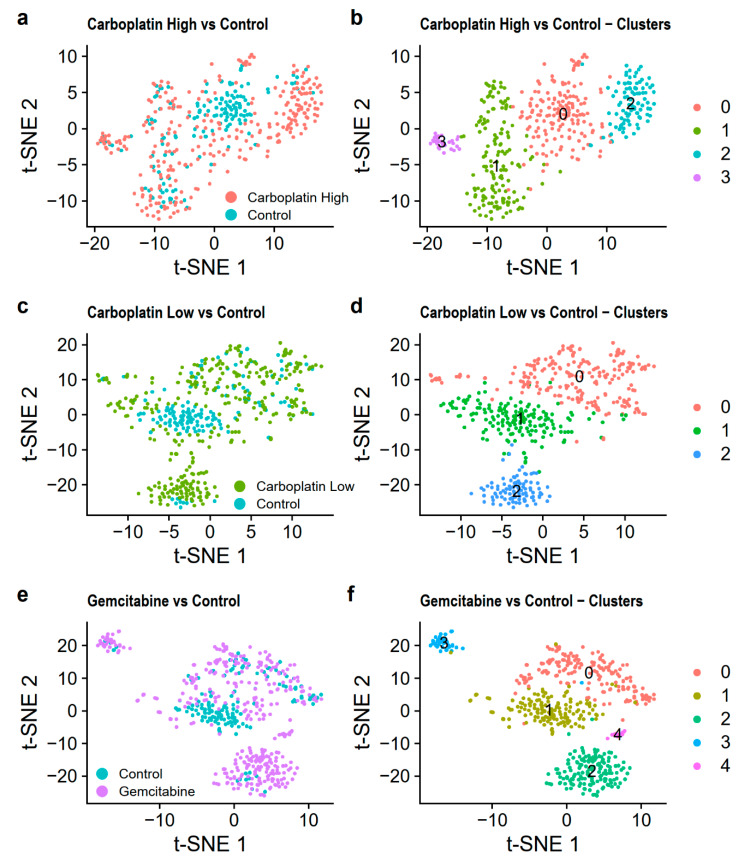
Visualization of the merged datasets using t-SNE. Carboplatin High vs. Control is shown colored (**a**) by the treatment and (**b**) by the identified clusters. Carboplatin Low vs. Control is shown colored (**c**) by the treatment and (**d**) by the identified clusters. Gemcitabine vs. Control is shown colored (**e**) by the treatment and (**f**) by the identified clusters.

PCA showed no clear clustering of the cells in the three merged datasets based on the first two PCs, Figure S5. The clustering then identified 4 clusters for Carboplatin High vs. Control, 3 clusters for Carboplatin Low vs. Control, and 5 clusters for Gemcitabine vs. Control. This was visualized using t-SNE in Figure 6 and UMAP in Figure S6. From this finding, it was evident that most of the control cells were found in clusters 0, 1, and 1, respectively, for Carboplatin High vs. Control, Carboplatin Low vs. Control, and Gemcitabine vs. Control. The number of cells in each cluster is listed in Table 4.

Differential gene expression was then compared between the treated and control cells within clusters 0 and 1 of the three merged datasets (the other clusters were not investigated since they included too few control cells, Table 4). This was done to see if we could find genes affected by the treatments in cells that are similar. Doing this, we found, on average, 193 differentially expressed genes after correction for multiple tests when comparing treated and control cells in clusters 0 and 1 of Carboplatin High vs. Control, Carboplatin Low vs. Control, and Gemcitabine vs. Control, all listed in Table S4. We then analyzed the enrichment of differentially expressed genes in KEGG pathways and GOs. All enrichments significant after correction for multiple testing are listed in Table S5. Some of the interesting findings were that the differences between Carboplatin High treated cells and Control cells within cluster 0 (Figure 6a,b) seems to be attributable to cancer-related KEGG pathways with the “p53 signaling pathway”, “longevity regulating pathway”, and “viral carcinogenesis”. The differences for cluster 1 lay mainly within the regulation of leukocytes through the GOs found among the top enrichments (adjusted *p* ≤ 0.01) “regulation of leukocyte chemotaxis”, “myeloid leukocyte migration”, “leukocyte chemotaxis”, “regulation of leukocyte migration”, and “leukocyte migration”. We did not find as many enrichments for Carboplatin Low vs. Control. This may be because we did not have enough cells from both samples in each cluster or because the treatment is not harsh enough to induce effects that are distinguishable after only 24 h of treatment. Gemcitabine vs. Control showed no enrichment in cluster 1, however, cluster 0 had enriched GOs and KEGG pathways, which indicates differences in immune cell response/activation through the GOs’ “response to molecule of bacterial origin”, “response to bacterium”, “regulation of symbiosis, encompassing mutualism through parasitism”, and “regulation of myeloid cell differentiation”, and the KEGG pathways “kaposi sarcoma-associated herpesvirus infection”, “salmonella infection”, “IL-17 signaling pathway”, “TNF signaling pathway”, and “apoptosis”.

## 4. Discussion

Advances in gene-expression analysis have recently come to the single-cell domain through bulk RNA sequencing with the rapid implementation of various scRNA-seq methodologies and protocols [11]. These methods have been applied to a variety of cells, but analyses comparing treated and control cells are few. As these methods are new, there is to date no gold-standard protocol for analyzing and interpreting the data in a standardized manner. This study shows how treated HSPCs and scRNA-seq can detect transcriptional differences induced by chemotherapeutic treatment through a comparison with control cells. We also provide general advice while proving the potential of the method for detecting transcriptional effects, which can be exploited in future studies of chemotherapy-induced toxicity in relevant cells types.

While there are many programs for the analysis of scRNA-seq data, our choice fell on the Seurat [23,24] R toolkit for single-cell genomics mainly due to its superior documentation and many implementations. We used both t-SNE [28] and UMAP [29] implemented in Seurat [23,24] for cluster visualization. We focus on the graphical representation of t-SNE in the present manuscript, while UMAP can be viewed in the supplement. T-SNE is the most widely used technique for scRNA-seq visualization, even though the newer UMAP is faster. UMAP is equally as good as t-SNE at local structures and even better for global structures [29]. For our reasonably small datasets, t-SNE’s longer computing times was not a major concern for us as the computing times were still just a couple of minutes long.

While interpreting the data, we found clear clusters both within the samples in Carboplatin High, Carboplatin Low, Gemcitabine, and Control, and when comparing the treated samples with the control in Carboplatin High vs. Control, Carboplatin Low vs. Control, and Gemcitabine vs. Control. The analysis of treated samples yielded more clusters, which indicates that the treatments induced considerable effects. However, one should note that the lower number of high-quality cells in the control sample, 157 compared to, on average, 338 in the treated samples, could prevent the algorithm from clustering rarer populations in the control sample. We recommend obtaining >300 high-quality cells. Using the Bio-Rad/Illumina ddSEQ™ setup, one could use two wells/sample to likely get >500 cells instead of just one well, which in the present study yielded, on average, 293 (157–390) high-quality cells. Another alternative would be to use another instrument, for example, the Chromium setup from 10X Genomics, which extracts many more cells/sample. However, as we can show that differences can be elucidated using only 300 cells/sample which also does not need as much sequencing as higher cell numbers would, the Bio-Rad/Illumina ddSEQ™ setup is, at least in our case, a more cost-effective setup at the moment.

From the differential expression analysis, we identified differentially expressed genes and enriched KEGG pathways, and GOs analysis exemplified key differences between clusters. Taking this a step further, we compared the control and treated cells that clustered together to find differences induced by the treatments. We could show induced changes attributable to the treatment at least in the clusters in which we had many cells from both treated and control samples. The fact that the treated cells also yielded more clusters indicates differences induced by the treatments. Consequently, we could also see that, when comparing Carboplatin Low vs. Control, there were fewer KEGG pathways and GOs than for the other samples. This milder treatment option also yielded fewer clusters in the integrated analysis compared to the other treatments. This finding indicates that the treatment must be sufficiently harsh (at least for these 24 h treatments) to be able to find treatment-induced effects. Consequently, we recommend using treatments at or around IC_50_ or longer exposure times for weaker treatments.

We are aware that our study only used cells from one sample and that the number of cells was quite low, at least for the Control sample. Therefore, we do not highlight the findings of specific genes and pathways (albeit significant after multiple testing) that differ between clusters and treatment conditions in the present study other than to show that our findings are within relevant cellular systems that could be important for HSPCs and chemotherapeutic treatments. Further, this also means that we have not in-depth analyzed specific cell type or lineage markers, for example, the ones presented by Laurenti et al. [32] and Novershtern et al. [33], among the cells and cluster, which is an important step to take in future studies. However, this methods-oriented study proves that scRNA-seq of HSPC is a feasible high-resolution approach for investigating the myelosuppressive effects of chemotherapeutic agents.

## 5. Conclusions

To conclude, the presented study shows that combining scRNA-seq and chemotherapy-treated HSPCs is a feasible approach for finding genes, pathways and biological processes that are affected by chemotherapeutic treatment, both within treated samples and when comparing treated and control cells. Further, we recommend obtaining >300 high-quality cells/sample and using a treatment close to IC_50_ to ensure that treatment effects can be captured. This study indicates the potential gains of using single-cell toxicity studies to find new personalized medicine tools for preventing and understanding toxicity.

## Figures and Tables

**Table 1:** Final concentrations of gemcitabine (ng/mL) and carboplatin (µg/ml) in the MTT assay.

Drug	Dilution Factor	1	2	3	4	5	6	7	8	9
**Gemcitabine**	4.00	47,619.05	11,904.76	2976.19	744.05	186.01	46.50	11.63	2.91	0.73
**Carboplatin**	4.00	1227.27	306.82	76.70	19.18	4.79	1.20	0.30	0.07	0.02

**Table 2:** High-quality cells and their gene expression.

	Number of High-Quality Cells	Average Number of Genes/Cell	*BCR*	*ABL1*	*BCR* and *ABL1*	*CD34*
**Carboplatin High**	290	1550	32	31	2	93
**Carboplatin Low**	335	2178	45	51	7	141
**Gemcitabine**	390	1313	37	34	8	76
**Control**	157	903	10	16	10	30

**Table 3:** The 25 most variable genes for Carboplatin High, Carboplatin Low, Gemcitabine, and Control.

Carboplatin High	Carboplatin Low	Gemcitabine	Control
*PF4*	*HBB*	*PF4*	*HBB*
*THBS1*	*PF4*	*PPBP*	*PF4*
*HBB*	*THBS1*	*CXCL8*	*PLEK*
*MEST*	*CLC*	*MPO*	*CD36*
*IGKC*	*HBA2*	*BTG2*	*THBS1*
*F13A1*	*CXCL8*	*HBB*	*TMSB4X*
*CXCL8*	*PLEK*	*HBA2*	*HBA2*
*PLEK*	*CA1*	*THBS1*	*MMRN1*
*PPBP*	*GP9*	*ATF3*	*F13A1*
*HBD*	*PPBP*	*GDF15*	*TOP2A*
*TMSB4X*	*HBD*	*JUN*	*SH3BP5*
*ISCA1*	*AHSP*	*F13A1*	*VWF*
*CXCL2*	*MPO*	*SAT1*	*F2R*
*CDKN1A*	*GPX1*	*TUBB1*	*PECAM1*
*GP9*	*CXCL3*	*CD69*	*FYB*
*MYLK*	*F13A1*	*GP9*	*LTB*
*MMRN1*	*CXCL2*	*CXCL1*	*HGD*
*LGALSL*	*MS4A1*	*CA1*	*HBG2*
*HBBP1*	*RNASE2*	*EGR1*	*CPA3*
*MDM2*	*VWF*	*CDKN1A*	*PPBP*
*CXCL3*	*CDKN1A*	*HBG2*	*HEXIM1*
*HGD*	*UBE2C*	*CXCL5*	*UBE2C*
*HPSE*	*HBBP1*	*LGALSL*	*CENPF*
*TUBB1*	*HPSE*	*IGKC*	*GPX1*
*GAS1*	*HGD*	*HPSE*	*DAB2*

**Table 4:** The number of cells in the identified clusters.

Cluster	Carboplatin High	Carboplatin Low	Gemcitabine	Control	Carboplatin High vs. Control	Carboplatin Low vs. Control	Gemcitabine vs. Control
**0**	130	183	198	98	167 (74, 93)	222 (170, 52)	178 (128, 50)
**1**	92	85	148	59	156 (106, 50)	175 (80, 95)	161 (70, 91)
**2**	68	67	30	-	96 (88, 8)	95 (85, 10)	154 (144, 10)
**3**	-	-	14	-	28 (22, 6)	-	40 (34, 6)
**4**	-	-	-	-	-	-	14 (14, 0)

## Supplementary Materials

The following are available online at https://www.mdpi.com/2073-4425/11/5/549/s1. Figure S1: Cumulative fraction of reads in samples, Figure S2: Visualization of the standard data pre-processing in Seurat, Figure S3: PCA separate samples, Figure S4: Sample cluster visualization using UMAP, Figure S5: PCA merged samples, Figure S6: Merged sample cluster visualization using UMAP, Table S1. Sequencing results and gene expression from Drop-seq, Table S2: Differentially expressed genes comparing sample clusters, Table S3: Enriched KEGG and GOs comparing sample clusters, Table S4: Differentially expressed genes comparing treatment vs. control, Table S5: Enriched KEGG and GOs comparing treatment vs. control.
