# Supplementary material for: Single-Cell RNA Sequencing of Hematopoietic Stem and Progenitor Cells Treated with Gemcitabine and Carboplatin
Source: Genes (Basel). 2020 May 14;11(5):549. doi: 10.3390/genes11050549 (PMC7288450; doi:10.3390/genes11050549)
Supplement: Supplementary file 1 [file genes-11-00549-s001.zip › Figures S1-S6.docx]

# **Supplementary Figures**

**
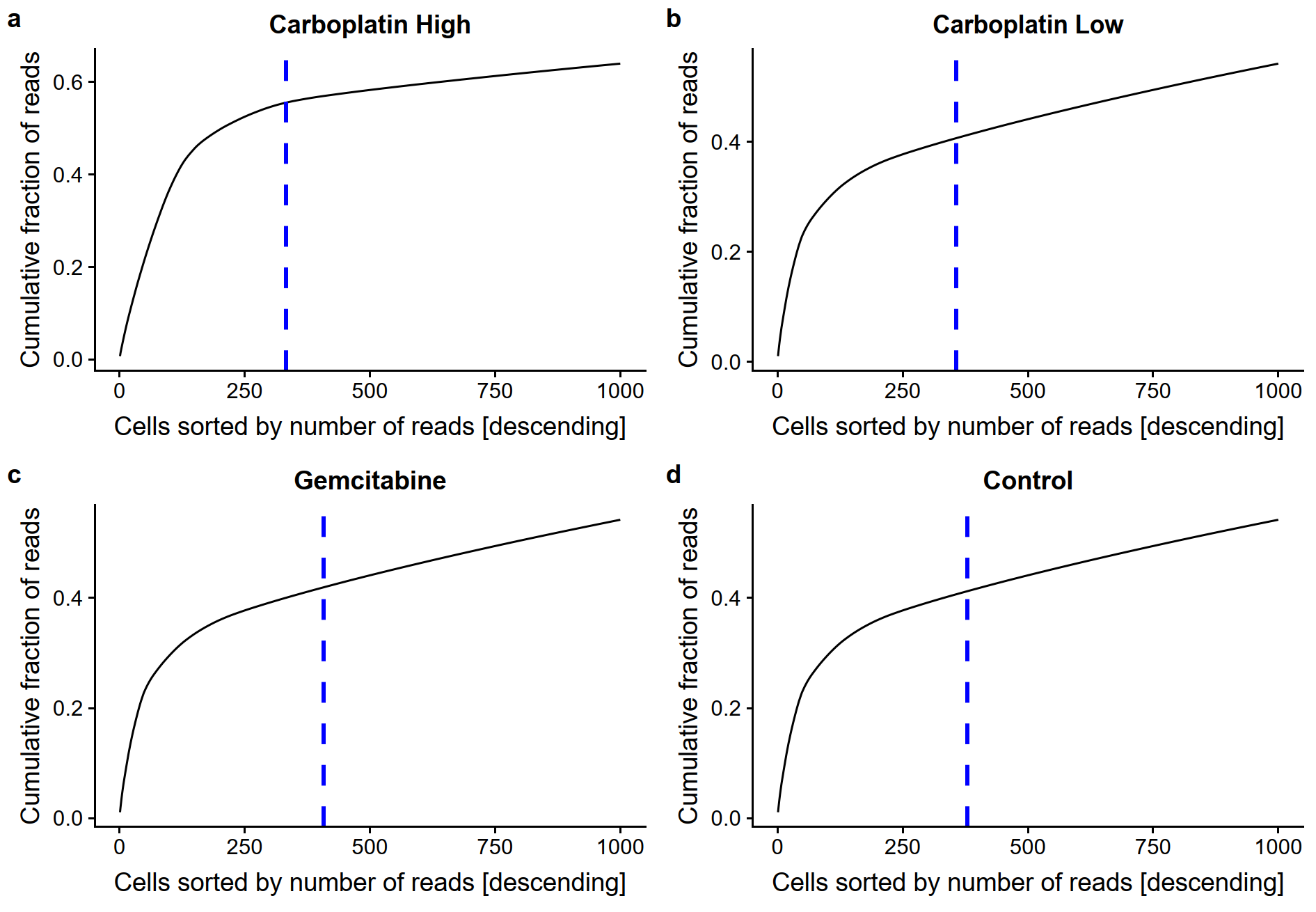
**

**Figure S1.** **Cumulative fraction of reads in samples.** Shows the cumulative fraction of reads from the cells sorted on the number of reads per cell in descending order. The blue lines correspond to when the fractional gain of reads dips below 0.025% when adding another cell. This yielded for a) **Carboplatin High** 332 cells, b) **Carboplatin Low** 357 cells, c) **Gemcitabine** 407 cells, and d) **Control** 379 cells. For these cells, the gene expression was determined using DigitalExpression in Drop-seq.

**
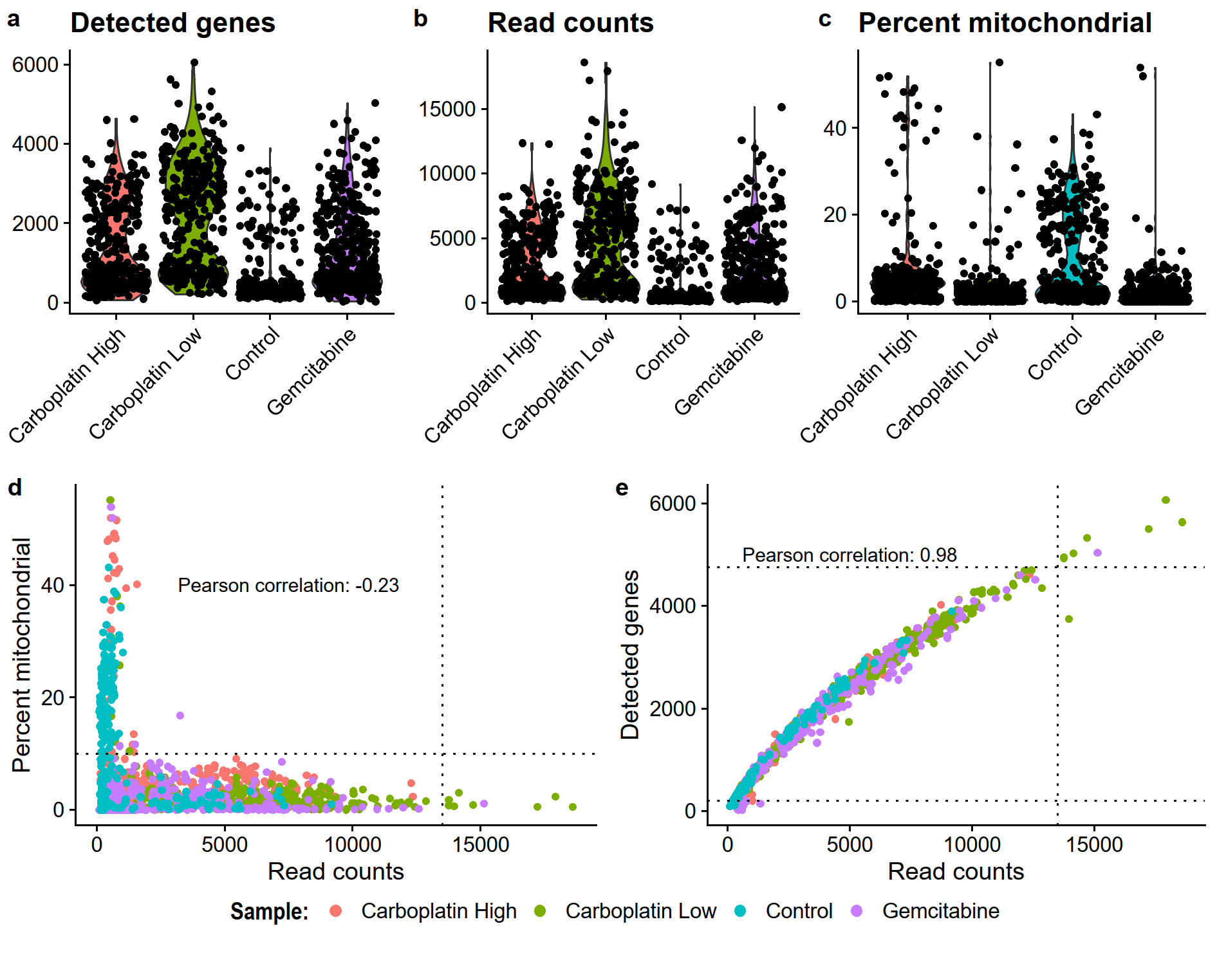
**

**Figure S2. Visualization of the standard data pre-processing in Seurat.** a) Shows the number of genes/cell in each sample. b) Shows the number of reads/cell in each sample. c) Shows the percentage of mitochondrial genes/cell in each sample. d) Shows the percent of mitochondrial genes against the read counts for all cells. e) Shows the number of detected genes against the read counts for all cells.

Based on this, cells with ≥10 % mitochondrial genes (above the horizontal dashed line in d)), ≥13500 reads/cell (to the right of the vertical dashed line in d) and e)), ≤200 genes/cell (below the lower horizontal dashed line in e)), or ≥4750 genes/cell (above the upper horizontal dashed line in e)) were filtered out.

**
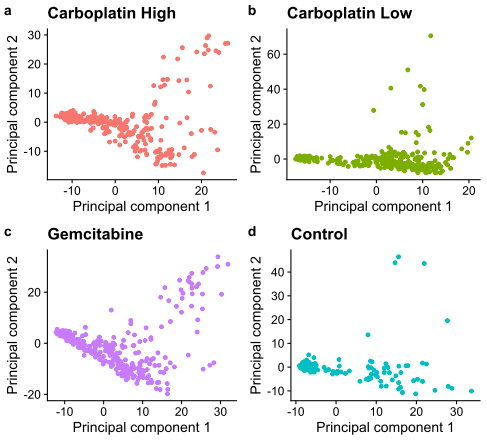
**

**Figure S3. PCA separate samples.** Shows the two first PCs of the cells in the four samples a) **Carboplatin High**, b) **Carboplatin Low**, c) **Gemcitabine**, and d) **Control**. No clear clustering was seen, however, some cells deviate from the mass on these two first PCs.

**
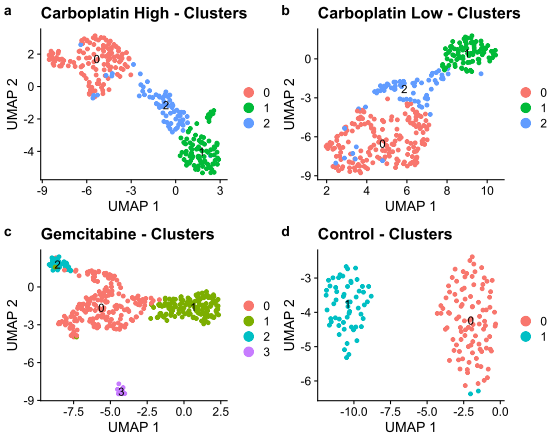
**

**Figure S4. Sample cluster visualization using UMAP.** Visualization colored by the identified clusters in the four samples a) **Carboplatin High**, b) **Carboplatin Low**, c) **Gemcitabine**, and d) **Control**.

**
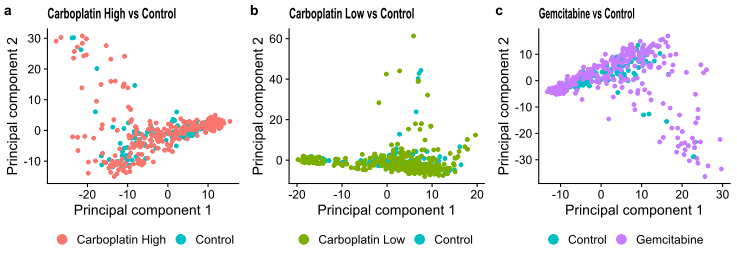
**

**Figure S5.** **PCA merged samples.** Shows the two first PCs of the cells in the merged datasets a) **Carboplatin High** **vs Control**, b) **Carboplatin Low** **vs Control**, and c) **Gemcitabine vs Control**. No clear clustering was seen, however, some cells separate from the majority.

**
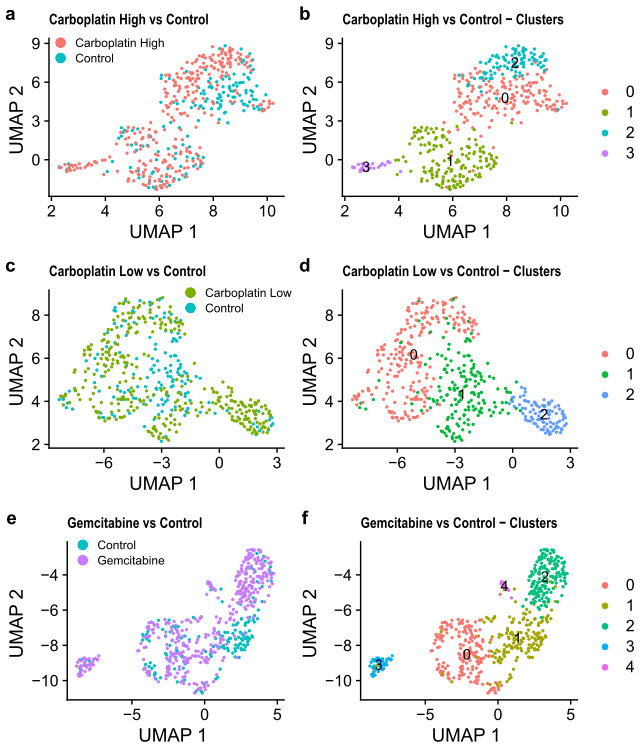
**

**Figure S6.** **Merged sample cluster visualization using UMAP.** **Carboplatin High** **vs Control** is shown colored by the treatment in a) and by the identified clusters in b). **Carboplatin Low** **vs Control** is shown colored by the treatment in c) and by the identified clusters in d). **Gemcitabine vs Control** is shown colored by the treatment in e) and by the identified clusters in f).
